# Modified mRNA as a new therapeutic option for pediatric respiratory diseases and hemoglobinopathies

**DOI:** 10.1186/s40348-015-0022-6

**Published:** 2015-11-20

**Authors:** Justin S. Antony, Alexander Dewerth, Ashiqul Haque, Rupert Handgretinger, Michael S.D. Kormann

**Affiliations:** Department of Pediatrics I-Pediatric Infectiology and Immunology, Translational Genomics and Gene Therapy in Pediatrics, University of Tübingen, Tübingen, Germany

**Keywords:** Modified mRNA, Pediatric diseases, Gene therapy

## Abstract

**Background:**

The immunogenicity and limited stability of conventional messenger RNA (mRNA) has traditionally restricted its potential therapeutic use. In 1992, the first clinical application of mRNA was reported as a potential protein-replacement therapy; however, subsequent investigations have not been made for almost two decades. Recent developments, including increased stability, controlling immunogenicity, as well as utilization of mRNA encoding zinc-finger nucleases (ZFNs), transcription activator-like effector nucleases (TALENs), and CRISPR-Cas9, have implicated modified mRNA as a very promising option for cancer immunotherapy, vaccines, protein expression replacement, and genome editing. This review aims to offer a summary of our present understanding of and improvements in mRNA-based drug technologies, along with a focus on the role in therapeutic options for pediatric respiratory diseases and hemoglobinopathies.

**Conclusions:**

This mini review summarizes the recent advances in modified mRNA-based therapy and its potential therapeutic effect in treating major pediatric diseases.

## Findings

### Introduction

Pediatric respiratory illness is a major cause of mortality and morbidity among infants and young children as they are more susceptible to respiratory diseases [1]. Asthma, tuberculosis, bronchiectasis, and bronchopulmonary dysplasia are chronic respiratory diseases in children. In addition, congenital respiratory disorders such as cystic fibrosis and primary ciliary dyskinesia are observed at lower incidence in children [2, 3]. Next to the lung diseases, hemoglobinopathies are the most common genetic disorders in pediatrics as at least 60,000 severely affected children are born every year [4, 5]. Although treatments are available for both clinical pictures, the major issue appears to be its limited effectiveness, thus providing only a short-term cure [5, 6]. However, recent advancements in science have paved the way to treat such diseases at the molecular level as new therapeutic targets and pathways are uncovered. In this review, we focus on common pediatric respiratory illness and hemoglobinopathies that are potentially amenable to gene therapy.

**Fig. 1:**
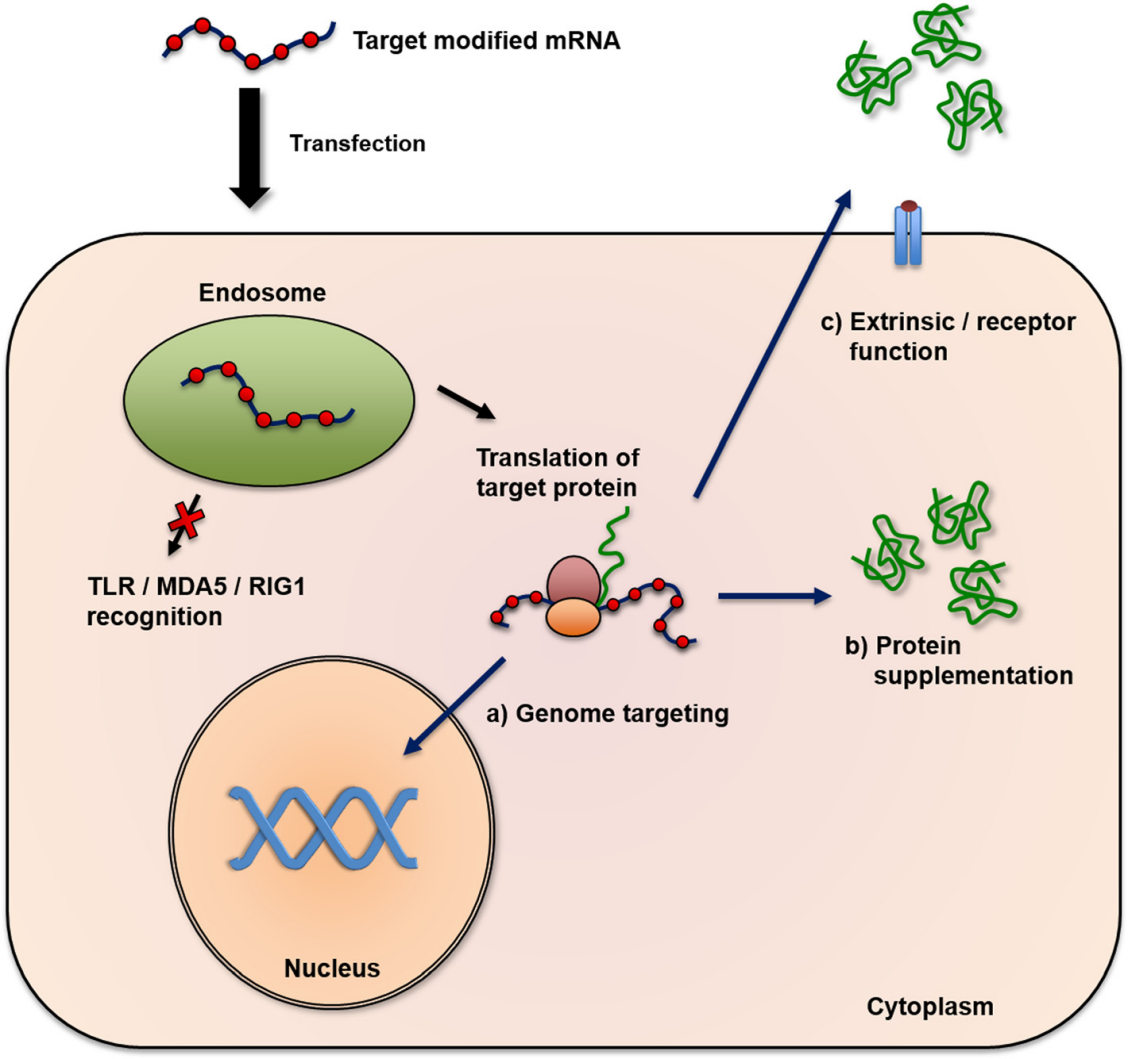
The principle and applications of modified mRNA. Modified mRNA can be transfected via several routes to the target cell, e.g., intraperitoneally, intravenously, or intratracheally. After endocytosis, the incorporation of naturally occurring, noncanonical nucleosides (indicated as *red dots*) into in vitro transcribed mRNA prevents activation of and consequently degradation by endosomal (Toll-like receptors, TLRs) and cytoplasmatic (MDA5, RIG1) mRNA-Sensors, thus being efficiently translated into a functional protein at the cell’s ribosomes. Different target applications can be applied for modified mRNA: (*a*) genome targeting, resulting in functional nucleases (e.g., zink-finger nucleases, CRISPR/Cas9) that bind and create sequence-specific double-strand breaks within the genome to facilitate gene correction, gene replacement or gene knock-out; (*b*) protein supplementation, where endogeneous protein malfunction can be overcome by restoring normal protein function; (*c*) extrinsic/receptor function in case of improper cell signaling

For two decades, gene therapy has been focused on plasmid DNA and viral DNA, with severe consequences in early key studies [7]. However, gene therapy has greatly evolved and has become a major focus in nucleotide-based gene therapy. Karikó et al. and Kormann et al. demonstrated that chemical modification of messenger RNA (mRNA) resulted in mRNA transcripts being less immunogenic and more efficiently translated in vitro [8] and in vivo [9]. Such chemically modified messenger RNA (mod. mRNA) has many advantages compared to other therapeutic nucleic acids. The most important features comprise a transient protein expression, reduced immunogenicity, superior translation efficiency, and pharmaceutical safety, as mod. mRNA does not integrate into the host genome [8, 10, 11]. Current progress in targeted genome editing mediated by nucleases and encoded by mod. mRNA to express engineered nucleases such as the CRISPR-Cas9 system, zinc-finger nucleases (ZFNs), and TAL effector nucleases (TALENs) will undoubtedly translate basic research findings to novel molecular therapeutics in the treatment of pediatric diseases [12, 13]. The principle and applications of mod. mRNA are illustrated in Fig. 1.

### Mod. mRNA: a better tool for gene therapy?

In a landmark publication, Warren et al. implicated chemically modified mRNA as a powerful tool to express proteins of interest in target cells in vitro. These authors showed that repeated administration of mod. mRNA encoding the Yamanaka factors KLF4, c-MYC, OCT4, and SOX2 could reprogram terminally differentiated human fibroblasts into pluripotent myofibroblasts without the need for retroviral vectors [14]. Consequently, the study provided an efficient way to reprogram cell fate without the risk of potential genomic integration. Subsequent studies have extended these initial observations of mod. mRNA-encoded protein expression to in vivo models, demonstrating specific expression in organs such as the heart, skeletal muscle, and lung [9, 15, 16].

### Mod. mRNA: reduced immunogenicity and increased stability

To achieve a beneficial therapeutic effect, reduced activation of innate immune receptors and an increased biological stability are key issues that need to be addressed when considering future clinical applications of mod. mRNA. Chemical substitutions such as the replacement of uridine by pseudouridine (Ψ) or cytosine by 5-methyl-cytosine (m5C) on target mRNA ultimately allow them to evade innate immune responses such as Toll-like receptor (TLRs) signaling pathways [8]. A study by Kormann et al. demonstrated that combining chemical modifications, such as the replacement of 25 % of uridine and cytidine with 2-thiouridine (s2U) and m5C reduced recognition of target mRNA through pattern recognition receptors, including TLR3, TLR7, TLR8, and RIG-I in human PBMCs. In addition, the same modifications also facilitated in vivo delivery of the respective mRNA [9]. These findings were verified by a subsequent study and confirmed that chemical modification effects in reduced innate immune recognition [17]. Very recently, it has been shown that the incorporation of N^1^-methylpseudouridine (m1Ψ) in mRNA resulted in innate immune evasion and increased translational capacity in vitro and in vivo [18]. Next to chemical modifications, other approaches to enhance stability and translational efficacy of mRNA include the use of phosphorothioate 5′ cap analogs, HPLC purification, and polyadenylation tail by a defined number of adenosines as well as the incorporation of stabilizer proteins [19–22]. The progress in lipid nanoparticle mediated delivery of mod. mRNA into target cells in vivo by various routes provided an efficient translation [23].

#### Nuclease-encoding mRNA: a novel strategy for permanent cure

**Fig. 2:**
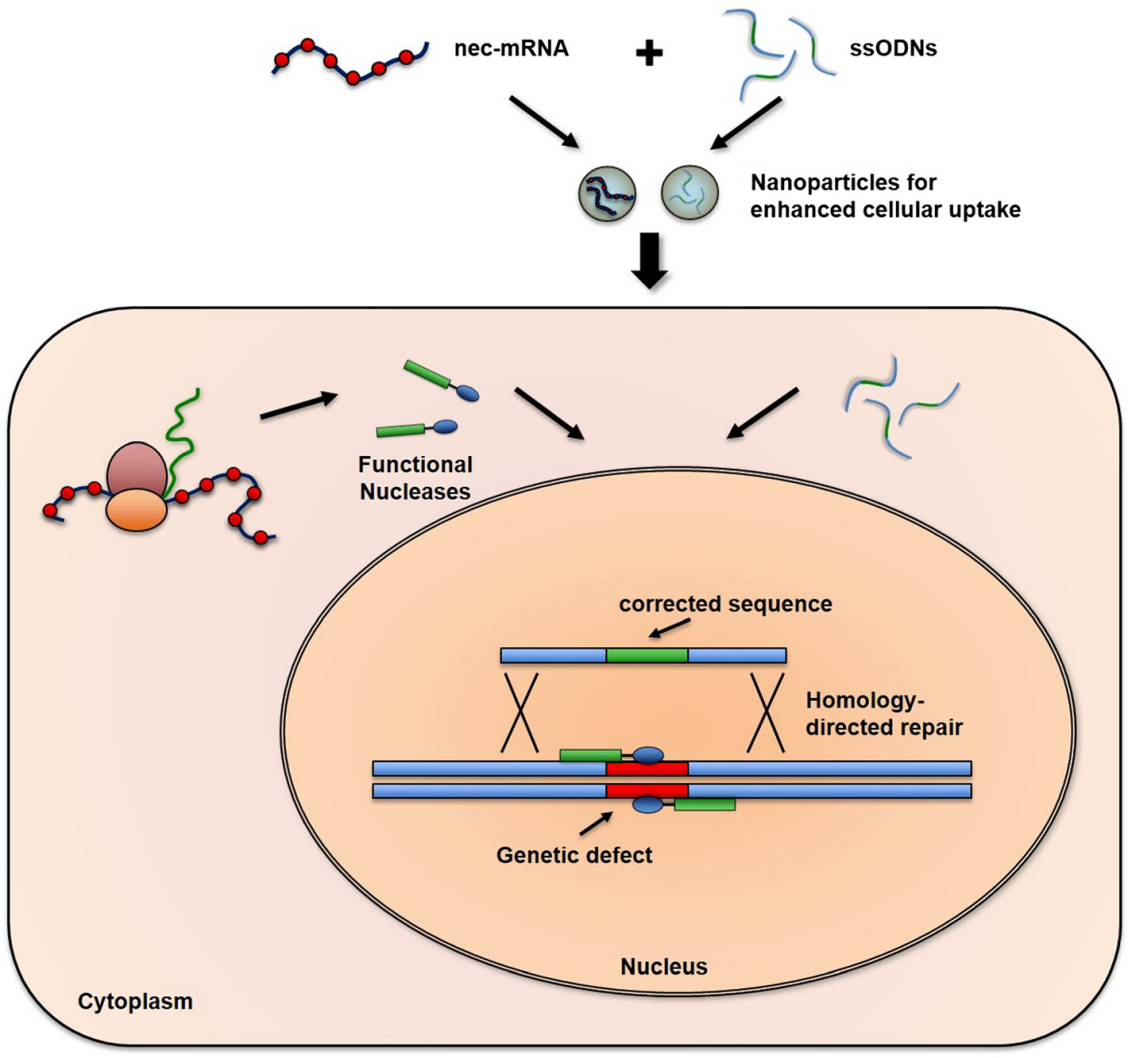
SNP correction using nec-mNRA and ssODNs. To facilitate site-specific gene correction of single nucleotide polymorphisms (SNPs), nec-mRNA and single-stranded oligodeoxynucleotides (ssODNs) can be efficiently administered to target cells (e.g., lung cells) by complexing both into positively charged nanoparticles for enhanced cellular uptake. Once in the cell, nec-mRNA gets translated into a functional nuclease that subsequently translocates to the nucleus and binds at sequence-specific sites next to the SNP. Generating a double-strand break (DSB), the genomic mutation can be corrected in the presence of ssODNs by means of homology-directed repair, resulting in proper gene function

Due to the transient expression of mod. mRNA, its use will be difficult in the treatment of chronic genetic diseases, as they require longer-term solutions. To circumvent this, **n**uclease-**e**ncoded **c**hemically mod. mRNA (nec-mRNA) has been described as a novel delivery paradigm. Expression of mRNA encoding zinc-finger nucleases (ZFNs) or transcription activator-like effector nucleases (TALENs) resulted in successful genome editing and subsequently the correction of surfactant protein B (SP-B) deficiency in mice [13]. The principle of nec-mRNA is illustrated in Fig. 2. Due to the rapid yet transient burst of expression of nec-mRNA, off-target effects will be minimized when compared to long-term expression by viral or plasmid vectors. In addition, the expression kinetics can be fine-tuned by different combinations of chemical modifications [24]. Thus, the proposed strategy might provide permanent gene correction by targeting stem cells. Moreover, the use of single-stranded oligodeoxynucleotides (ssODNs)—short, single-stranded DNA-based repair templates—for gene targeting of short nuclear polymorphisms (SNPs) would completely abrogate the need for viral vectors, resulting in patient-specific gene correction and thus an ultimate cure.

### Proof of principle: use of mod. mRNA to treat pediatric diseases

Cystic fibrosis, SP-B deficiency, asthma, β thalassemia, and sickle cell anemia are all genetic diseases that can affect children. With its potential for protein replacement and gene correction, mod. mRNA presents a powerful tool to minimize or even cure these conditions. The next part of the review will focus on the present evidence of positive results using mod. mRNA against genetic disease.

#### SP-B deficiency

Surfactant protein B (SP-B) is a pulmonary surfactant protein that reduces surface tension and prevents the collapse of alveoli. Congenital SP-B deficiency is a rare, lethal, and monogenic disease induced mostly by loss-of-function mutations on both *SFTPB* alleles. The administration of surfactants, corticosteroids, and other immunosuppressants, repeated lung lavage, and ultimately lung transplantation are the only therapeutic interventions currently available. However, these treatments show poor efficacy and outcome [25]. In mice, repeated administration of mod. SP-B mRNA by intratracheal high-pressure spraying significantly increased SP-B protein levels, resulting in the elimination of pulmonary inflammation and a significant increase in survival [9]. Furthermore, a single intratracheal application of nec-mRNA combined with AAV-encoded repair template increased the life span of SP-B deficient mice due to genetic correction in alveolar type II cells [13].

#### Cystic fibrosis

Cystic fibrosis (CF) is the most life-limiting monogenic disease in Caucasian populations and is caused by a mutation in the gene that encodes the cystic fibrosis transmembrane and conductance regulator (CFTR). CF is characterized by altered epithelial mucus secretion in several organs, most severely in the pulmonary epithelium [26]. In spite of the various clinical trials performed with viral and nonviral vector-mediated gene therapy approaches in CF patients, these techniques have shown low efficacy [27]. Therefore, a viable alternative therapy has yet to be developed, e.g., via repeated administration of therapeutic nucleic acids that penetrate the mucus of CF patients. Modified mRNA has already shown great potential to increase protein expression in the lungs [9]. It is estimated that only 10 % of normal levels of CFTR activity is sufficient to avoid the disintegrating effects seen in CF [28] making mod. mRNA a promising tool to restore adequate CFTR expression in the lungs. In addition, the transfection of chemically modified CFTR mRNA in mutated CFBE41o− cells restored cAMP-induced CFTR currents similar to wild type cells due to the mRNA-driven replacement of functionally active channels [20].

### Pediatric asthma

Asthma is the most common chronic inflammatory disorder in childhood and is associated with airway hyper-responsiveness leading to recurrent episodes of wheezing, breathlessness, chest tightness, coughing, and airflow obstruction. However, in children below 5 years of age, clinical symptoms of asthma differ individually in a nonspecific manner. Usually, inhalation of corticosteroids and bronchodilators is the first choice when it comes to the treatment of asthma. However, some patients face the clinical picture of corticosteroid resistance as a consequence to repeated corticosteroid administration. In this case, treatment by means of gene replacement could be an alternative route and possibly benefit these patients. One such target for gene therapy would be to influence Th2 cytokine reactions [29] by inducing regulatory T cells. Mays et al. demonstrated that administration of modified *Foxp3* mRNA in a time- and site-specific fashion in murine lungs prevented allergic asthma in vivo, while suppressing Th2 responses [15].

#### β-Thalassemia and sickle cell disease

As one of the most common single-gene defects across the globe, β-thalassemia represents a significant burden for affected patients and families. Driven by various mutations in the β-globin gene cluster, the disease is characterized by a reduction or absence of β-globin gene expression. Ultimately, this results in a lack of functional adult hemoglobin (HbA), which is composed of two alpha- and two beta-globin chains (α_2_β_2_) [4]. Without sufficient hemoglobin, red blood cells develop abnormally, leading to severe anemia. This imbalance in α- and β-globin production also hinders erythroid precursor maturation, resulting in ineffective erythropoiesis.

Mutations in the human hemoglobin beta (*HBB*) gene cause β-thalassemia and those that are homozygous for a given mutation suffer severe anemia. In children, anemia begins to develop within the first month of life and infants fail to thrive. Currently, allogeneic bone marrow transplantation and hematopoietic stem cell transfusion are the only available curative schemes; in turn, these treatments are limited to a minority of patients due to the availability of histocompatible donors. However, gene therapy based on autologous transplantation of genetically corrected hematopoietic stem cells (HSCs) shows high potential as a cure, since it is not restricted to histocompatible donors and immunosuppression. The lentiviral delivery of a normal HBB gene into hematopoietic stem cells could be shown to result in therapeutic benefit [30]. However, viral vectors always possess the risk of causing insertional mutagenesis. Recent developments in gene correction using nec-mRNA encoding proteins such as ZFNs, TALENS, and CRISPR-Cas9 have shown high potential to effectively correct genes while mitigating the danger of internal mutagenesis. It has been reported that using CRISPR-Cas9 (with modified mRNA) to cleave the HBB gene and *piggyBac* for homologous recombination selection can correct two different β-thalassemia mutations and thus reduce the genetic status to a heterozygous state, where patients are only mildly anemic and capable of leading normal lives [31].

Sickle cell disease is caused by mutations in the protein-coding *HBB gene*. Replacement of A to T results in valine instead of glutamic acid. Both copies of the gene need to contain this particular mutation to cause anemia. A recent report suggests that seamless *HBB* gene correction is possible using TALENs and *piggyBac* [32]. Together with its precise correcting efficiency and high modularity, these nucleases represent a promising tool to correct such a SNP [33].

### Future directions

mRNA has tremendous potential for both gene therapy and gene correction approaches. However, as with every new technology, a number of methodological improvements and specific challenges need to be addressed. The chemical modification needs to be adapted to the target cell type and to the transfection reagent used. In addition, the incorporation of chemically modified nucleosides into mRNA increases protein stability but decreases translational efficiency [24]. Thus, it is recommended that mRNA formulations undergo extensive optimization to achieve a therapeutic benefit. Furthermore, in order to achieve permanent cures for genetic diseases as that mentioned above, the nec-mRNA and repair templates must be directed to stem/progenitor cells by targeted cell therapy.

In conclusion, the therapeutic potential of mod. mRNA has been rapidly recognized for the treatment of pediatric diseases. However, further improvements in systemic delivery, consideration of different cell types or different organs, and effective chemical modifications for each such type are pivotal for efficient utilization. Combining efforts to overcome cell turnover together with appropriate animal models will enable the field to make continued progress and to reach the step into the clinic.
